# Does providing more services increase the primary hospitals’ revenue? An assessment of national essential medicine policy based on 2,675 counties in China

**DOI:** 10.1371/journal.pone.0190855

**Published:** 2018-01-16

**Authors:** Fei Chen, Min Yang, Qian Li, Jay Pan, Xiaosong Li, Qun Meng

**Affiliations:** 1 West China School of Public Health, Sichuan University, Chengdu, Sichuan, China; 2 West China Research Centre for Rural Health Development, Sichuan University, Chengdu, Sichuan, China; 3 Centre for Health Information and Statistics, National Health and Family Planning Commission of China, Beijing, China; Dartmouth-Hitchcock Medical Center, UNITED STATES

## Abstract

**Objective:**

To understand whether the increased outpatient service provision (OSP) brings in enough additional income (excluding income from essential medicine) for primary hospitals (INCOME) to compensate for reduced costs of medicine.

**Methods:**

The two outcomes, annual OSP and INCOME for the period of 2008–2012, were collected from 34,506 primary hospitals in 2,675 counties in 31 provinces in China by the national surveillance system. The data had a four-level hierarchical structure; time points were nested within primary hospital, hospitals within county, and counties within province. We fitted bivariate five-level random effects regression models to examine correlations between OSP and INCOME in terms of their mean values and dose-response effects of the essential medicine policy (EMP). We adjusted for the effects of time period and selected hospital resources.

**Findings:**

The estimated correlation coefficients between the two outcomes’ mean values were strongly positive among provinces (r = 0.910), moderately positive among counties (r = 0.380), and none among hospitals (r = 0.002) and time (r = 0.007). The correlation between their policy effects was weakly positive among provinces (r = 0.234), but none at the county and hospital levels. However, there were markedly negative correlation coefficients between the mean and policy effects at -0.328 for OSP and -0.541 for INCOME at the hospital level.

**Conclusion:**

There was no evidence to suggest an association between the two outcomes in terms of their mean values and dose-response effects of EMP at the hospital level. This indicated that increased OSP did not bring enough additional INCOME. Sustainable mechanisms to compensate primary hospitals are needed.

## Introduction

In response to allegations that it is too difficult and too expensive for many Chinese people to seek health care, China’s central government launched a new round of national healthcare reform in April 2009. The main goals of this reform included accelerating the construction of the basic medical security system, establishing national essential medicine policy (EMP), improving the primary medical and health service system, promoting the equality of basic public health services and promoting government-run hospitals reform [1]. The EMP, an essential part of the new round of national healthcare reform, aimed to improve availability, affordability, quality and safety of essential medicine. It requested government-run primary hospitals to use low-cost medicine with zero profit [2–4]. The EMP had been experimental since 2009; it scaled up to all government-run primary hospitals at the end of 2011, which include the urban-based community health centers and rural-based township and town center hospitals [5–6]. The EMP was implemented among the hospitals in a temporal sequence from 2009 to 2012; no hospital was on the policy in 2008, and there were 27.6%, 26.4%, 25.8% and 20.2% hospitals exposed to the policy each year from 2009 to 2012 respectively.

Numerous studies have shown some positive effects of the policy, such as the increased service usage due to reduced costs of medicine [7–9], reduced medicine costs in treatment [9–12], increased accessibility to affordable medicines [13], and rational drug use [9,13–15]. However, some scholars reported some contrary results, such as decreased availability of lowest-priced generic medicines [3], lack of access to essential paediatric medicine [16], remaining irrational use of antibiotics [17] and ineffective compensation for health-care providers [9].

One of the main effects of EMP was to reduce the cost of medicines. This would certainly weaken the hospitals’ financial situations [10, 18–20]; under the EMP, the income of primary health care providers came mainly from the medical service charges and government subsidies [21]. However, the system of government subsidies was not sound [22–24]. As a result, many primary hospitals had to bring in income from medical services to cover their staff salaries; hence an increasing medical services was a way to compensate for the reduced cost of medicine. Those services generated income from medical tests and clinical treatment in addition to medicines. We believed that increased OSP services were less likely generating much income for compensation purpose for several reasons. Firstly, under the current medical insurance scheme many treatments and tests could not be reimbursed if they were provided as outpatient services, and hence less likely be accepted by patients; Secondly, increasing OSP was also known a positive effect of EMP since the reduced costs of medicine simulated the usage of outpatient services [7–9]; Thirdly, costs of medicine and treatment per service were reduced under EMP [7–9]. Hence compensation for the reduced costs of medicine would mostly come from inpatient services. The outcome INCOME that excluded costs of medicines would reflect mostly incomes of hospitals brought in by medical tests and inpatient services. If the EMP effect was reflected by the increased OSP services, the on-going compensation mechanism could be reflected by an increased hospital income. An interesting research question would be the correlation between the change trend of OSP and that of INCOME at hospital level. A positive correlation between change trends of OSP and INCOME could indicate the compensation mechanism at such level. To date, few scholars have studied this problem at a national level. One reason could be lack of effective methods in the typical policy evaluation field to handle the complexity of the data, which have a four-level hierarchical structure; time points are nested within primary hospital, hospitals within county, and counties within province. Clustering effects among provinces, counties and hospitals introduce dependence in the data, which is not appropriately analyzed using conventional methods, such as difference-in-differences (DID) [25]. However, a multivariable multilevel model, well-developed and widely applied in social sciences research, can effectively handle these kinds of data to answer our research questions [26]. In light of this background, the purpose of this study is to examine the correlations between OSP and INCOME at different levels with a bivariate multilevel model.

## Materials and methods

### National Health Resource and Medical Service Survey (NHRMSS)

NHRMSS was a national survey that was conducted among all healthcare-related facilities every year from 2008–2012 in all of China to acquire the basic information about facilities for the new healthcare reform. The survey was conducted by the Center for Health Information and Statistics, National Health and Family Planning Commission of China [27]. The survey instrument for primary hospitals collected data at the end of the year. It included questions on number of staff, hospital beds, housing and basic construction, quantity of large equipment, income and expenditure, assets and liabilities, medical service and preventive primary public health service. This study used data from 34,506 primary hospitals (township hospitals (TH), township central hospitals (TCH) and community health service centres (CHC)) in 2,675 counties of 31 provinces in China. Located in rural counties, the THs and TCHs provide basic health care primarily for rural population. In contrast the CHCs are located in districts of cities and center of some big counties. They provide primary health care mainly for city population living in the catchment areas. Context information, such as consumer price index (CPI), was obtained from the China Statistical Yearbook [28]. The basic data was presented in Tables 1 and 2:

**Table 1:** Description of the data structure.

Hospital	Hospital type	County*	Province*	Region	Year	TETP**	OSP(times)***	INCOME(¥)***
1	CHC	110113	11	East	2008	0	10.294	15.658
1	CHC	110113	11	East	2009	0	10.287	15.681
1	CHC	110113	11	East	2010	0	10.348	15.895
1	CHC	110113	11	East	2011	0	10.453	15.885
1	CHC	110113	11	East	2012	1	10.454	16.235
┊	┊	┊	┊	┊	┊	┊	┊	┊
34506	CHC	652324	65	West	2008	0	8.987	12.653
34506	CHC	652324	65	West	2009	0	8.768	12.322
34506	CHC	652324	65	West	2010	1	8.738	13.306
34506	CHC	652324	65	West	2011	2	8.907	14.181
34506	CHC	652324	65	West	2012	3	8.757	13.878

* Administrative area code;

**Time exposed to policy in year;

***Logarithm transformed value.

**Table 2:** Description of main variables.

Variables	Variable interpretation	Number (%)
Hospital	Number of hospitals	34,506(100)
Hospital type		
CHC	Community health service center	2,218(6.4)
TH	Township hospitals	22,777(66.0)
TCH	Township central hospitals	9,511(27.6)
Region		
East	East part of China	8,983(26.0)
Middle	Middle part of China	10,375(30.1)
West	West part of China	15,148(43.9)
Year		
2008	No intervention	34,506(20)
2009	The first year of intervention	34,506(20)
2010	The second year of intervention	34,506(20)
2011	The third year of intervention	34,506(20)
2012	The fourth year of intervention	34,506(20)
Hospital by time exposed to policy (year)		
0	No exposure	83,766(48.6)
1	One-year policy exposure	34,506(20.0)
2	Two-year policy exposure	26,791(15.5)
3	Three-year policy exposure	18,201(10.5)
4	Four-year policy exposure	9,266(5.4)
OSP(times)**		*Mean (SD)*
2008	OSP of 2008	9.44(1.19)
2009	OSP of 2009	9.53(1.20)
2010	OSP of 2010	9.54(1.21)
2011	OSP of 2011	9.56(1.23)
2012	OSP of 2012	9.68(1.24)
INCOME(**¥**)**		*Mean (SD)*
2008	INCOME of 2008	13.5(1.26)
2009	INCOME of 2009	13.7(1.25)
2010	INCOME of 2010	14.0(1.21)
2011	INCOME of 2011	14.3(1.18)
2012	INCOME of 2012	14.5(1.16)

** Mean & SD after logarithm transformation.

### Basic information variables

Six hospital-level measures are: (1) time exposed to policy of hospital in year (TETP) ranged from 0 to 4 years, (2) location (eastern, central and western regions of China) and hospital type, (3) quantity of staff, (4) quantity of health technical personnel, (5) quantity of bed space, and (6) total assets. These variables showed the basic features of the hospital in terms of staff, assets and equipment, and properly reflected the characteristics of the hospital. Dividing measures (4) and (5) by measure (3), we obtained two new variables: health technical personnel ratio (HTPR) and bed space ratio (BSR), respectively, to measure relative quality of human resource and relative treatment bed space. In addition, dividing measure (6) by measure (3), the logarithm transformed into the new variable, total asset ratio (LTAR), to reflect a relative capacity in medical tests or diagnosis.

### Outcome variables

Two main outcomes at the hospital level are: (1) number of outpatient service provision (OSP) and (2) total income, excluding income from essential medicine (INCOME). INCOME was adjusted by the nation’s CPI in each year against that in 2012, and logarithm transformation was performed on both OSP and INCOME due to their skewed distributions.

### Statistical analysis

We were interested in four questions. Firstly, were OSP and INCOME correlated overall, and if so, in what direction? Secondly, were there dose-response effects (or change trends) of the EMP for OSP and INCOME overall? If so, it could demonstrate the general intensity and sustainability of the EMP. Thirdly, would the correlations and the dose-response effects remaining after adjusting for covariates? Fourthly, were OSP and INCOME correlated in terms of their mean values and dose-response effects of the EMP at corresponding levels, respectively? This could remind us whether the two outcomes followed the same trend as the EMP.

Based on the data structure, previous research had applied four-level repeated measures models to assess dose-response effects of the EMP policy for single outcome, such as OSP [29]. For correlation of two outcomes, multivariate multilevel models were constructed by creating an extra level “below” the original level 1 units to define the multivariate structure [30]. Thus, to answer the first question, we first fitted a bivariate, five-level null model to examine correlations between OSP and INCOME in terms of their mean values. Then, to answer the second question, we fitted a bivariate, five-level model with independent variable TETP to identify the dose-respond effects of the EMP. Thirdly, we fitted a bivariate, five-level model with explanatory variables to answer the third question, adjusting effects of time period and some hospital resources, which included HTPR, LTAR, the location and the type of the hospital. And last, we fitted bivariate, five-level random effects regression models to answer the fourth question. We used MLwiN software to conduct the multilevel analysis [31].

## Results

### Bivariate, five-level null model for unadjusted overall correlation between OSP and INCOME

We fitted a bivariate, five-level null model to obtain the raw correlation relationship between OSP and INCOME at different levels of the data structure without adjusting other influential factors. Specific definitions for the hierarchy included:

1. Level 5: provinces, denoted by m;
2. Level 4: counties, denoted by l;
3. Level 3: hospitals, denoted by k;
4. Level 2: time, denoted by j;
5. Level 1: indicator to define the bivariate structure, denoted by r;

Details of the model were introduced as following:

$$\begin{array}{ll} resp_{rjklm} & =I(r=1)\times const\times \beta_{0}+I(r=2)\times const\times \beta_{1}\\ & +I(r=1)\times (g_{0m}+f_{0lm}+v_{0klm}+u_{0jklm})+I(r=2)\times (g_{1m}+f_{1lm}+v_{1klm}+u_{1jklm}) \end{array}$$

$$\left[\begin{array}{l} g_{0m}\\ g_{1m} \end{array}\right]\sim N(0,\Omega_{g}):\Omega_{g}=\left[\begin{array}{ll} \sigma_{g0}^{2} & \\ \sigma_{g01} & \sigma_{g1}^{2} \end{array}\right]\;\left[\begin{array}{l} f_{0lm}\\ f_{1lm} \end{array}\right]\sim N(0,\Omega_{f}):\Omega_{f}=\left[\begin{array}{ll} \sigma_{f0}^{2} & \\ \sigma_{f01} & \sigma_{f1}^{2} \end{array}\right]\;\left[\begin{array}{l} v_{0klm}\\ v_{1klm} \end{array}\right]\sim N(0,\Omega_{v}):\Omega_{v}=\left[\begin{array}{ll} \sigma_{v0}^{2} & \\ \sigma_{v01} & \sigma_{v1}^{2} \end{array}\right]\;\left[\begin{array}{l} u_{0jklm}\\ u_{1jklm} \end{array}\right]\sim N(0,\Omega_{u}):\Omega_{u}=\left[\begin{array}{ll} \sigma_{u0}^{2} & \\ \sigma_{u01} & \sigma_{u1}^{2} \end{array}\right]$$

The terms *resp*_1*jklm*_, *resp*_2*jklm*_ stood for OSP and INCOME respectively; while *β*_*0*_, *β*_*1*_ were the regression coefficients of constant item *const* (value equal to 1) for the two outcomes. Meanwhile, *g*_0*m*_, *f*_0*lm*_, *v*_0*klm*_, and *u*_0*jklm*_ were residual of *const* at levels five, four, three and two for OSP, and the same for INCOME. *β*_*0*_ and *β*_*1*_ were the mean values of OSP and INCOME, and they were the fixed coefficients of the model.

In addition, Ω_*g*_, Ω_*f*_, Ω_*v*_ and Ω_*u*_ were variance and covariance matrices for residuals of units at levels five, four, three and two respectively. Following the definition of Pearson’s correlation, elements in these matrices could be used to calculate correlation relationships between OSP and INCOME at corresponding levels, and they were the random coefficients of the model.

The estimate of fixed and random parameters was presented in Table 3.

**Table 3:** The estimate value of bivariate five-level null model.

Effects	Parameters	Coefficients (standard error)	χ^2^	*P*
Fixed effects	*β*_*0*_ (constant)	9.676(0.154)	3947.8	<0.000
	*β*_*1*_ (constant)	14.156(0.182)	6049.8	<0.000
Random effects				
level 5 (province)	$\sigma_{g0}^{2}$	0.729(0.188)	15.0	<0.001
	*σ*_*g*01_	0.821(0.216)	14.4	<0.001
	$\sigma_{g1}^{2}$	1.018(0.261)	15.2	<0.001
level 4 (county)	$\sigma_{f0}^{2}$	0.324(0.011)	867.6	<0.000
	*σ*_*f*01_	0.148(0.008)	342.3	<0.000
	$\sigma_{f1}^{2}$	0.366(0.012)	930.3	<0.000
level 3 (hospital)	$\sigma_{v0}^{2}$	0.541(0.005)	11707.2	<0.000
	*σ*_*v*01_	−0.006(0.003)	4.0	0.046
	$\sigma_{v0}^{2}$	0.413(0.004)	10660.6	<0.000
level 2 (time)	$\sigma_{u0}^{2}$	0.202(0.001)	40840.0	<0.000
	*σ*_*u*01_	0.036(0.001)	1296.0	<0.000
	$\sigma_{u0}^{2}$	0.356(0.001)	126736.0	<0.000

The Pearson’s correlation coefficient between the two outcomes was calculated based on the estimated variance-covariance in Table 3 for each level in the data respectively as follows:

$$\text{Among}\;\text{provinces}:\;r_{5}=\frac{0.821}{\sqrt{0.729\times 1.018}}=0.953;\;\text{Among}\;\text{counties}:\;r_{4}=\frac{0.148}{\sqrt{0.324\times 0.366}}=0.430$$

$$\text{Among}\;\text{hospitals}:\;r_{3}=\frac{-0.006}{\sqrt{0.541\times 0.413}}=-0.013;\;\text{Among}\;\text{time}\;\text{points}:\;r_{2}=\frac{0.036}{\sqrt{0.202\times 0.356}}=0.134$$

Based on the model estimates we obtained a strong positive association between mean values of OSP and INCOME among 31 provinces, moderately positive among 2,675 counties, and weak among 34,506 facilities and time points. Testing the significance of the covariance between the two outcomes (*σ*_*g*01_, *σ*_*f*01_, *σ*_*v*01_, *σ*_*u*01_) at the four levels jointly using a generalized Wald test, we obtained *χ*^2^ test statistic with with *P* values as less than 0.000, less than 0.000, 0.046 and less than 0.000 respectively, as shown in the last column in Table 3. This suggested that the two outcomes’ correlation at corresponding levels had statistical significance. More out-patient services brought more income to the facilities across provinces and among counties are generally expected as hospital performance. Further question of analysis is whether dose-response effects of the EMP of the two outcomes were also correlated among counties and facilities in particular, to examine possible compensation mechanism.

### Bivariate, five-level model for fixed dose-response effects and effects of independent variables

#### Dose-response effects of the EMP

We first brought an independent variable, hospital by time exposed to policy (TETP), into the null model to identify the raw dose-response effects of the EMP. The fixed part of the model was as follows, and the estimated values are presented in Table 4. The random effects in relation to residuals at all levels remained unchanged from the last model.

$$\begin{array}{ll} resp_{rjklm} & =I(r=1)\times const\times \beta_{0}+I(r=2)\times const\times \beta_{1}+I(r=1)\times TETP\times \beta_{2}+I(r=2)\times TETP\times \beta_{3}\\ & +I(r=1)\times (g_{0m}+f_{0lm}+v_{0klm}+u_{0jklm})+I(r=2)\times (g_{1m}+f_{1lm}+v_{1klm}+u_{1jklm}) \end{array}$$

*β*_*2*_ and *β*_*3*_ were the mean values of dose-effects of EMP for OSP and INCOME respectively, and they reflected the strength and direction of EMP effects.

**Table 4:** The estimate value of dose-effect model.

Effects	Parameters	Coefficient (standard error)	χ^2^	*P*
**Fixed effects**	*β*_*0*_ (constant)	9.610(0.154)	3894.1	<0.000
	*β*_*1*_ (constant)	13.843(0.181)	5849.3	<0.000
	*β*_*2*_	0.064(0.001)	4096.0	<0.000
	*β*_*3*_	0.310(0.001)	961.0	<0.000
**Random effects**				
level 5 (province)	$\sigma_{g0}^{2}$	0.731(0.187)	15.3	<0.001
	*σ*_*g*01_	0.820(0.214)	14.7	<0.001
	$\sigma_{g1}^{2}$	1.010(0.258)	15.3	<0.001
level 4 (county)	$\sigma_{f0}^{2}$	0.320(0.011)	846.3	<0.000
	*σ*_*f*01_	0.137(0.008)	293.3	<0.000
	$\sigma_{f1}^{2}$	0.361(0.012)	905.0	<0.000
level 3 (hospital)	$\sigma_{v0}^{2}$	0.541(0.005)	11707.2	<0.000
	*σ*_*v*01_	*0.000(0.003)	0.024	0.877
	$\sigma_{v0}^{2}$	0.473(0.004)	13983.1	<0.000
level 2 (time)	$\sigma_{u0}^{2}$	0.196(0.001)	38416.0	<0.000
	*σ*_*u*01_	0.008(0.001)	64.0	<0.000
	$\sigma_{u0}^{2}$	0.219(0.001)	47961.0	<0.000

*0.00048(0.00307)

The results showed that dose-effects of EMP for OSP and INCOME were positive and significant, with *P* values both less than 0.000, demonstrating both increased OSP and INCOME as the exposure time to the EMP policy increased. The inclusion of the TETP variable reduced the -2*log likelihood from 691297.7 down to 624794.3 to give a much improved model showing significant effects of the dose-response trends. Meanwhile, the correlations between the two outcomes had no obvious changes.

#### Adjusting for effects of time period and hospital resources

We introduced other explanatory variables—year (comparing to year 2008), HTPR, LTAR, hospital type (comparing to CHC), region (comparing to East), the first order interaction between TETP and hospital type, and the first order interaction between TETP and region—into the model to adjust the confounders, and then we identified the correlation relationship between OSP and INCOME at different levels and the true dose-response effects of the EMP. The estimated values are presented in Table 5.

$$\begin{array}{ll} resp_{rjklm} & =I(r=1)\times const\times \beta_{0}+I(r=2)\times const\times \beta_{1}+I(r=1)\times TETP\times \beta_{2}+I(r=2)\times TETP\times \beta_{3}+(I(r=1)+I(r=2))\times X\times \beta_{x}\\ & +I(r=1)\times (g_{0m}+f_{0lm}+v_{0klm}+u_{0jklm})+I(r=2)\times (g_{1m}+f_{1lm}+v_{1klm}+u_{1jklm}) \end{array}$$

*X* stood for the other explanatory variables, and *β*_*x*_ were their regression coefficients.

**Table 5:** The estimate of model after adjusting for effects of time period and hospital resources.

Effects	Parameters	OSP			INCOME		
		*β (SE)*	χ^2^	*P*	*β (SE)*	χ^2^	*P*
**Fixed effects**	*Const*(*β*_*0*_, *β*_*1*_)	9.560(0.173)	3053.7	<0.000	14.460(0.208)	5849.3	<0.000
	TETP(*β*_*2*_, *β*_*3*_)	0.044(0.005)	77.4	<0.000	-0.052(0.004)	961.0	<0.000
	HTPR	0.309(0.013)	565.0	<0.000	-0.029(0.012)	5.8	0.016
	LTAR	0.033(0.001)	1089.0	<0.000	0.008(0.001)	64.0	<0.000
	Hospital type						
	TH	-0.125(0.025)	25.0	<0.000	-0.672(0.021)	1024.0	<0.000
	TCH	-0.129(0.026)	24.6	<0.000	0.218(0.021)	107.8	<0.000
	Region						
	Middle	-0.620(0.285)	4.7	0.030	-0.597(0.348)	2.9	0.089
	West	-0.791(0.188)	17.7	<0.000	-0.914(0.224)	16.6	<0.000
	The first order interaction between TETP and hospital type						
	TETP with TH	-0.017(0.004)	18.1	<0.000	0.072(0.004)	324.0	<0.000
	TETP with TCH	-0.017(0.005)	11.6	<0.001	0.024(0.004)	36.0	<0.000
	The first order interaction between TETP and region						
	TETP with Middle	-0.016(0.003)	28.4	<0.000	-0.004(0.003)	1.8	0.180
	TETP with West	-0.024(0.003)	64.0	<0.000	0.004(0.002)	4.0	0.046
	Year						
	2009	0.074(0.003)	608.4	<0.000	0.230(0.003)	5877.8	<0.000
	2010	0.075(0.004)	351.6	<0.000	0.480(0.004)	14400.0	<0.000
	2011	0.084(0.005)	282.2	<0.000	0.818(0.005)	26765.0	<0.000
	2012	0.182(0.007)	676.0	<0.000	1.022(0.006)	29013.4	<0.000
**Random effects**		***β (SE)***			**χ**^**2**^		***P***
level 5 (province)	$\sigma_{g0}^{2}$	0.411(0.106)			15.0		<0.001
	*σ*_*g*01_	0.458(0.123)			13.9		<0.001
	$\sigma_{g1}^{2}$	0.621(0.159)			15.3		<0.000
level 4 (county)	$\sigma_{f0}^{2}$	0.308(0.010)			948.6		<0.000
	*σ*_*f*01_	0.131(0.008)			268.1		<0.000
	$\sigma_{f1}^{2}$	0.351(0.011)			1018.2		<0.000
level 3 (hospital)	$\sigma_{v0}^{2}$	0.523(0.004)			17095.6		<0.000
	*σ*_*v*01_	0.001(0.002)			0.25		0.617
	$\sigma_{v0}^{2}$	0.312(0.003)			10816.0		<0.000
level 2 (time)	$\sigma_{u0}^{2}$	0.194(0.001)			37636.0		<0.000
	*σ*_*u*01_	*0.003(0.000)			25.0		<0.000
	$\sigma_{u0}^{2}$	0.177(0.001)			31329.0		<0.000

*0.0025(0.0005)

After adjusting for the confounders, the estimated correlation coefficients between the mean values of OSP and INCOME were 0.906, 0.398, 0.002, and 0.014 at the four levels, with *P* values of the *χ*^2^ test statistic for the covariance at each of the four levels being less than 0.000, less than 0.000, 0.617, and less than 0.000 respectively. It suggested that the two outcomes’ correlation at province and county levels still had statistical significance. However, the regression coefficient of TETP for INCOME became negative (-0.052), while that of OSP was still positive (0.044). The negative effect could suggested that longer exposure time to the EMP policy could be associated with a decreased INCOME, and the positive one could suggested that a longer exposure to the EMP may trigger an increased outpatient services in general. These directions of changes fitted the objectives of the EMP policy. Besides, after including time period and hospital resources, the -2*log likelihood declined to 580308.2 from the dose-response model’s value of 624794.3, suggesting that this was a much improved model.

### Random coefficients model for TETP

At last, we fitted a random coefficients model for TETP. The model was as follows, and the results are presented in Table 6.

$$\begin{array}{l} \begin{array}{ll} resp_{rjklm} & =I(r=1)\times const\times \beta_{0}+I(r=2)\times const\times \beta_{1}+I(r=1)\times TETP\times \beta_{2}+I(r=2)\times TETP\times \beta_{3}+(I(r=1)+I(r=2))\times X\times \beta_{x}\\ & +I(r=1)\times (g_{0m}+f_{0lm}+v_{0klm}+u_{0jklm}+g_{2m}+f_{2lm}+v_{2klm})+I(r=2)\times (g_{1m}+f_{1lm}+v_{1klm}+u_{1jklm}+g_{3m}+f_{3lm}+v_{3klm}) \end{array} \end{array}$$

$$\left[\begin{array}{l} g_{0m}\\ g_{1m}\\ g_{2m}\\ g_{3m} \end{array}\right]\sim N(0,\Omega_{g}):\Omega_{g}=\left[\begin{array}{llll} \sigma_{g0}^{2} & & & \\ \sigma_{g01} & \sigma_{g1}^{2} & & \\ \sigma_{g02} & \sigma_{g12} & \sigma_{g2}^{2} & \\ \sigma_{g03} & \sigma_{g13} & \sigma_{g23} & \sigma_{g3}^{2} \end{array}\right]\;\left[\begin{array}{l} f_{0lm}\\ f_{1lm}\\ f_{2lm}\\ f_{3lm} \end{array}\right]\sim N(0,\Omega_{f}):\Omega_{f}=\left[\begin{array}{llll} \sigma_{f0}^{2} & & & \\ \sigma_{f01} & \sigma_{f1}^{2} & & \\ \sigma_{f02} & \sigma_{f12} & \sigma_{f2}^{2} & \\ \sigma_{f03} & \sigma_{f13} & \sigma_{f23} & \sigma_{f3}^{2} \end{array}\right]\;\left[\begin{array}{l} v_{0klm}\\ v_{1klm}\\ v_{2klm}\\ v_{3klm} \end{array}\right]\sim N(0,\Omega_{v}):\Omega_{v}=\left[\begin{array}{llll} \sigma_{v0}^{2} & & & \\ \sigma_{v01} & \sigma_{v1}^{2} & & \\ \sigma_{v02} & \sigma_{v12} & \sigma_{v2}^{2} & \\ \sigma_{v03} & \sigma_{v13} & \sigma_{v23} & \sigma_{v3}^{2} \end{array}\right]\;[\begin{array}{l} u_{0jklm}\\ u_{1jklm} \end{array}]\sim N(0,\Omega_{u}):\Omega_{u}=\left[\begin{array}{ll} \sigma_{u0}^{2} & \\ \sigma_{u01} & \sigma_{u1}^{2} \end{array}\right]$$

**Table 6:** The fitting results of two responses with five-level model after including the confounders.

Effects	OSP			INCOME		
	*β (SE)*	χ^2^	*P*	*β (SE)*	χ^2^	*P*
**Fixed effects**						
*Const*(*β*_*0*_, *β*_*1*_)	9.622(0.167)	3319.7	<0.000	14.506(0.211)	4726.4	<0.000
TETP(*β*_*2*_, *β*_*3*_)	0.041(0.017)	5.8	0.016	-0.059(0.024)	6.0	0.014
HTPR	0.291(0.013)	501.1	<0.000	-0.037(0.012)	9.5	0.002
LTAR	0.031(0.001)	961.0	<0.000	0.005(0.001)	25.0	<0.000
Hospital type						
TH	-0.142(0.026)	29.2	<0.000	-0.644(0.022)	856.9	<0.000
TCH	-0.146(0.027)	29.8	<0.000	0.251(0.023)	119.1	<0.000
Region						
Middle	-0.626(0.275)	5.2	0.023	-0.623(0.352)	3.1	0.078
West	-0.811(0.181)	20.1	<0.000	-0.917(0.225)	16.6	<0.000
The first order interaction between TETP and hospital type						
TETP with TH	0.002(0.007)	0.1	0.752	0.042(0.006)	49.0	<0.000
TETP with TCH	0.002(0.008)	0.1	0.752	-0.011(0.006)	3.7	0.054
The first order interaction between TETP and region						
TETP with Middle	-0.035(0.025)	2.0	0.157	0.036(0.037)	0.9	0.343
TETP with West	-0.038(0.021)	3.3	0.069	0.008(0.031)	0.1	0.752
Year						
2009	0.074(0.003)	608.4	<0.000	0.231(0.003)	5929.0	<0.000
2010	0.075(0.003)	625.0	<0.000	0.481(0.003)	25706.8	<0.000
2011	0.081(0.004)	410.1	<0.000	0.816(0.004)	41616.0	<0.000
2012	0.174(0.006)	841.0	<0.000	1.017(0.006)	28730.3	<0.000
**Random effects**						
Level 5 (province)	***β (SE)***			**χ**^**2**^		***P***
$\sigma_{g0}^{2}$	0.383(0.099)			15.0		<0.001
*σ*_*g*01_	0.450(0.121)			13.8		<0.001
$\sigma_{g1}^{2}$	0.683(0.164)			17.3		<0.000
*σ*_*g*02_	0.013(0.006)			4.7		0.030
*σ*_*g*12_	0.013(0.008)			2.6		0.107
$\sigma_{g2}^{2}$	0.003(0.001)			9.0		0.003
*σ*_*g*03_	-0.001(0.009)			0.01		0.920
*σ*_*g*13_	0.002(0.011)			0.03		0.862
*σ*_*g*23_	0.001(0.001)			1.0		0.317
$\sigma_{g3}^{2}$	0.006(0.002)			9.0		0.003
Level 4 (county)						
$\sigma_{f0}^{2}$	0.312(0.010)			973.4		<0.000
*σ*_*f*01_	0.131(0.008)			268.1		<0.000
$\sigma_{f1}^{2}$	0.381(0.012)			1008.1		<0.000
*σ*_*f*02_	-0.006(0.002)			9.0		0.003
*σ*_*f*12_	-0.002(0.002)			1.0		0.317
$\sigma_{f2}^{2}$	0.011(0.0005)			484.0		<0.000
*σ*_*f*03_	0.0005(0.002)			0.1		0.752
*σ*_*f*13_	-0.028(0.002)			196.0		<0.000
*σ*_*f*23_	0.001(0.0004)			6.3		0.012
$\sigma_{f3}^{2}$	0.022(0.001)			484.0		<0.000
Level 3 (hospital)						
$\sigma_{v0}^{2}$	0.579(0.005)			13409.6		<0.000
*σ*_*v*01_	0.001(0.003)			0.1		0.752
$\sigma_{v1}^{2}$	0.362(0.003)			14560.4		<0.000
*σ*_*v*02_	-0.037(0.001)			1369.0		<0.000
*σ*_*v*12_	*0.000 (0.001)			0.0004		0.984
$\sigma_{v2}^{2}$	0.022(0.0004)			3025.0		<0.000
*σ*_*v*03_	-0.0001(0.001)			0.01		0.920
*σ*_*v*13_	-0.023(0.001)			529.0		<0.000
*σ*_*v*23_	**0.000(0.000)			1.0		0.317
$\sigma_{v3}^{2}$	***0.005(0.000)			625.0		<0.000
Level 2 (time)						
$\sigma_{u0}^{2}$	0.152(0.001)			23104.0		<0.000
*σ*_*u*01_	****0.001(0.000)			6.3		0.012
$\sigma_{u1}^{2}$	0.146(0.001)			21316.0		<0.000

*0.00002(0.001);

**0.0002(0.0002);

***0.005(0.0001);

****0.001(0.0004).

The terms *β*_2_, *g*_2*m*_, *f*_2*lm*_ and *v*_2*klm*_ were the fixed parameter, residuals of TETP at level five unit, four unit, and three unit respectively for the dependent variable OSP. Similarly the corresponding terms for the dependent variable INCOME were *β*_3_, *g*_3*m*_, *f*_3*lm*_ and *v*_3*klm*_. The terms *β*_2_ and *β*_3_ were the mean values of TETP for dependent variables OSP and INCOME.

In addition, Ω_*g*_, Ω_*f*_, Ω_*v*_ and Ω_*u*_ were variance and covariance matrices for residual of *const* and TETP at level five unit, four unit, three unit and residual of *const* at level two unit, respectively. They reflected the correlation relationship between OSP and INCOME in terms of their mean values and dose-response effects of the EMP at corresponding levels. For example, the correlation between the two outcomes in term of their dose-response effects at the province level was calculated by *σ*_*g*23_, $\sigma_{g2}^{2}$ and $\sigma_{g3}^{2}$. Finally, the -2*log likelihood declined to 552211.6, which meant that the good fit was becoming better.

Based on the model estimates, the correlation coefficients of random intercepts between the two outcomes were remained similar as the previous models at all four levels. Based on random slope estimates, greater policy effects on OSP resulted in larger effects on INCOME (r = 0.234) among provincials, but none on the county and facility levels. In addition, the correlation coefficients of OSP between intercepts and slopes were moderately negative among facilities (r = -0.328), and none at the county level. The correlation coefficients of INCOME between intercepts and slopes were negative among counties (r = -0.306), among facilities (r = -0.541), and none at the province level. The results of this model indicated a number of possibilities in practice. Firstly, at provincial level the non-essential medical income decreased more as the exposure time to the EMP policy became longer, and a reversed trend effects of the out-patient services. And such change trends were correlated between the two outcomes. Second, counties with more out-patient services initially tended to have smaller effects of the EMP policy over time, and so was for the INCOME or non-essential medical revenues. Thirdly, facilities having higher INCOME initially tended to achieved less effects of the EMP policy over time.

## Discussion

Hospitals or healthcare facilities located in the same area are more similar in terms of number of staff, assets, scale and socio-economic environment, compared to hospitals in different areas. This phenomenon is called clustering [32] and is the main reason for regional difference of hospitals’ OSP and INCOME distribution. The multiple linear regression model is commonly used to analyze associations based on structured data. Such model, however, has limitations and cannot separate the environmental factors’ effects from the variance [33–34]. In contrast, the multilevel statistical models could decompose the total variance of the outcome variables into corresponding levels according to data hierarchy [35] and have been applied in a variety of fields [36–37]. The use of bivariate multilevel models enables us to effectively investigate the correlations between multiple dependent variables with stratification by level of the hierarchy in data [38].

For the first and third research questions, the results indicated that provinces with higher mean value of OSP tended to have higher mean value of INCOME, and the same relationship of the two outcomes among counties. Although there was no previous study that directly examined this relationship, it is possible that prescriptions of non-essential medicines, excessive examinations by advanced equipment at out-patient services could rise medical revenue for hospital income as a compensation mechanism for the revenue lost due to the zero-marked essential medicine. However, this correlation was not observed at the hospital or facility level where the motivation of compensation was present. Further research into the implementation of the EMP policy and its impact at the facility level is guaranteed.

For the second and third questions, we found positive dose-response effects for OSP, but negative dose-response effects for INCOME. The findings partly suggested the expected overall effects of the EMP policy, i.e., a longer exposure to the EMP policy was associated with an increased out-patient services overall, but partly a side effects of the financial issue for primary healthcare facilities, i.e., a reduced income associated with longer exposure to the policy. The positive effect of EMP on OSP was supported by some previous studies [7–9, 39]. A study in Hubei province found an obvious increasing in OSP after implementing the EMP [7]. A study of primary hospitals showed evidence of the EMP effects in terms of ‘two downs and one up”: outpatient charges and hospital expenses down, and OSP up [8]. A study by Li [9] presented an increase of outpatient (including emergency) visits to the township hospitals in Ningxia, Chongqing and Tianjin by 5.7%, 24.0% and 6.2% respectively, in the post-implementation period than in the pre-implementation period. Meanwhile, a study in Anhui province by Xu [39] concluded a 22.43% of increase in the average number of outpatient and emergency visits after implementation of the EMP. However, one study did not found the positive effect of EMP on OSP, but reported that the increase in OSP was largely due to over-prescription by many CHCs [19]. The negative effect of the EMP on INCOME found in this study was supported by another study [22] in which the author showed a drop of the overall income among 63.33% primary hospitals after the implementation of EMP in Hubei province. The negative effect might be induced by the following: The total income of primary hospitals consisted mainly of fiscal subsidies revenue, medical revenue and drug revenue [20], and government fiscal subsidies revenue was the basis for ensuring smooth implementation of EMP [21,40]. In our study, INCOME was made up of mainly fiscal subsidies revenue and medical revenue. Meanwhile, medical revenue actually increased to some extent because of increased services [7–9, 39]. Thus, inadequate subsidies revenue might be the main reason for the negative effect on INCOME, supported by the findings of other studies [8, 22]. Further study is required on government fiscal subsidies policies and how they were implemented at primary hospital level and impacted on the ‘true’ income of primary hospitals.

For the fourth question, we found that only at provincial level the policy effects on OSP and on INCOME was weakly positive among provinces (r = 0.234). This suggests that there was no evidence to support the hypothesis that effect of the EMP on OSP was linked with that on INCOME at facility or hospital level. Meanwhile, the results of third question were also indicated a lack of significant correlation between the mean values of OSP and INCOME at the hospital level. It is known that before implementing EMP, drug revenue was the main part of the primary hospitals’ total income [41]. Meanwhile, after implementing EMP, fiscal subsidies revenue and medical revenue were the two main parts of the primary hospitals’ total income [41]. There was a positive effect of EMP on OSP found in this study and many others, which meant that more and more people are choosing primary hospitals when they seek health care, and this phenomenon actually augments the medical revenue to some extent. However, the hypothesis that increased OSP brings in more INCOME to hospitals as compensation for reduced costs of medicine was not observed at the hospital level. It suggested to us that in general hospitals did not provide services or treatment more than necessary to compensate for the loss of the medicine sales, and compared to medical revenue, fiscal subsidies revenue was a relatively more important part of primary hospitals’ total income [20]. In addition, the markedly negative correlation coefficients between the mean and policy effects at -0.328 for OSP and -0.541 for INCOME at the hospital level suggested that hospitals with lower mean OSP or INCOME before the policy implementation tended to achieve greater policy effects, i.e. gaining both more OSP and INCOME over the policy exposure period.

Though there was no evidence to suggest an association between OSP and INCOME, in terms of both their means and policy effects at hospital level, the moderately strong association between the two outcomes at the province level could be the result of complex contextual effects. Further research to reveal the context factors and hospital services are required. The phenomenon that hospitals with lower mean value of OSP or INCOME showed greater change in gaining more OSP or INCOME warrants further research on the mechanism by micro-level factors.

## Conclusions

The EMP has a positive effect on OSP, but a negative effect on INCOME. There was no evidence to suggest an association between the two outcomes in terms of their mean values and dose-response effects of EMP at the hospital level, which indicated that increased OSP did not bring enough additional INCOME. To ensure smooth implementation of EMP, sustainable mechanisms to compensate primary hospitals by government are needed.

## Limitation

A number of limitations in the study should be reminded for the interpretation of the findings. Firstly, due to availability of the data, we did not have context variables at the levels of province and county to explain variabilities in the outcome variables at the corresponding levels. Second, we could not separate the true income revenue from subsidies ones in the INCOME variable. The total income of primary healthcare facilities came from a number of sources: medical revenue, fiscal subsidies, higher facilities subsidies and other incomes such as facility initiated complementary services for fees. The INCOME variable used in the study was the total income minus essential medical revenue. The higher facilities subsidies were usually small and variable among facilities, which had little impact on the finding. The finding of increased income in the study could be confounded with increasing of fiscal subsidies revenue. However, since the increasing of fiscal subsides was generally the same to all primary healthcare facilities nationwide, such effects would have be captured by the time effects in our model. The dose-response effects in relation to the policy should be unaffected. Thirdly, we used OSP, but the INCOME included outpatient and hospitalization parts; however, the medical income of primary hospitals came mainly from the outpatient part. Lastly, because the EMP was implemented in a temporal sequence, it led to different primary hospitals with different time exposed to the policy.
